# Out-of-the tropics or trans-tropical dispersal? The origins of the disjunct distribution of the gooseneck barnacle *Pollicipes elegans*

**DOI:** 10.1186/s12983-015-0131-z

**Published:** 2015-12-30

**Authors:** Sergio Marchant, Amy L. Moran, Peter B. Marko

**Affiliations:** Department of Biological Sciences, Clemson University, 132 Long Hall, Clemson, SC 29634 USA; Department of Biology, University of Hawai‘i at Mānoa, Honolulu, HI 96822 USA

**Keywords:** ABC, Climate, Coalescent, Demographic history, Diversity, Marine, Out-of-the tropics, Phylogeography, Sea surface temperature

## Abstract

**Background:**

Studying species with disjunct distributions allows biogeographers to evaluate factors controlling species ranges, limits on gene flow, and allopatric speciation. Here, we use phylogeographic and population genetic studies of the barnacle *Pollicipes elegans* to discriminate between two primary hypotheses about the origin of disjunct distributions of extra-tropical populations: trans-tropical stepping-stone colonization versus an out-of-the tropics origin.

**Results:**

Nucleotide diversity peaked in the centre of the species’ range in samples from El Salvador and was lower in samples from higher latitudes at Mexico and Peru. Haplotypes from El Salvador samples also had a deeper coalescent, or an older time to a most recent common ancestor. A deep phylogeographical break exists between Mexico and all samples taken to the south (El Salvador and Peru). Isolation-with-migration analyses showed no significant gene flow between any of the three regions indicating that the difference in genetic differentiation among all three regions is explained primarily by differences in population separation times. Approximate Bayesian Computation model testing found strong support for an out-of-the tropics origin of extra-tropical populations in *P. elegans*.

**Conclusions:**

We found little evidence consistent with a stepping-stone history of trans-tropical colonization, but instead found strong evidence for a tropical origin model for the largely disjunct distribution of *P. elegans*. Sea surface temperature and habitat suitability are likely mechanisms driving decline of populations in tropical regions, causing the disjunct distribution.

**Electronic supplementary material:**

The online version of this article (doi:10.1186/s12983-015-0131-z) contains supplementary material, which is available to authorized users.

## Background

Species with disjunct or geographically discontinuous ranges are important systems for understanding the factors controlling species’ distributions, population connectivity, and the process of allopatric speciation [1–10]. The two main biogeographic mechanisms that have been proposed to explain range disjunctions are dispersal across an uninhabitable region; or vicariance, the fragmentation of a species’ ancestral range through the formation of an unsuitable or impassable region. In both cases, allopatric speciation can take place if the gap in a species’ range sufficiently limits gene flow [11]. Although vicariance and dispersal may not be mutually exclusive processes [12–14], distinguishing their roles remains central to the overall goal of biogeography [12–18], to characterize the primary mechanisms that create spatial and temporal patterns of biodiversity [19].

Because dispersal and vicariance may be discriminated when the ages of both the barrier and the population separations are known [20], understanding the timing of divergence events is often key to understanding how disjunct distributions arise [21]. However, biogeographic tests based on the timing of population separation or speciation relative to known geological events rely fundamentally on estimates of mutation rates, which are often only known from related taxa and may depend on additional untestable assumptions. Furthermore, for many marine taxa, temporal inferences are further complicated by the fact that the formation of many barriers to dispersal in the sea are likely climate-driven and geologically short-lived, with limited information about their exact location and duration [22, 23]. However, genetic data can be used to test biogeographic hypotheses using other approaches that do not rely on molecular clock assumptions [24–26], but instead attempt to detect biogeographic processes that leave behind distinct neutral genetic diversity signatures. The potential pitfall of this alternative approach is that it cannot be used to reconstruct ancient biogeographic events [24] because the telltale population genetic signatures that they leave behind will be gradually erased by the evolutionary forces of mutation, gene flow, genetic drift, and natural selection [23, 27].

**Fig. 1:**
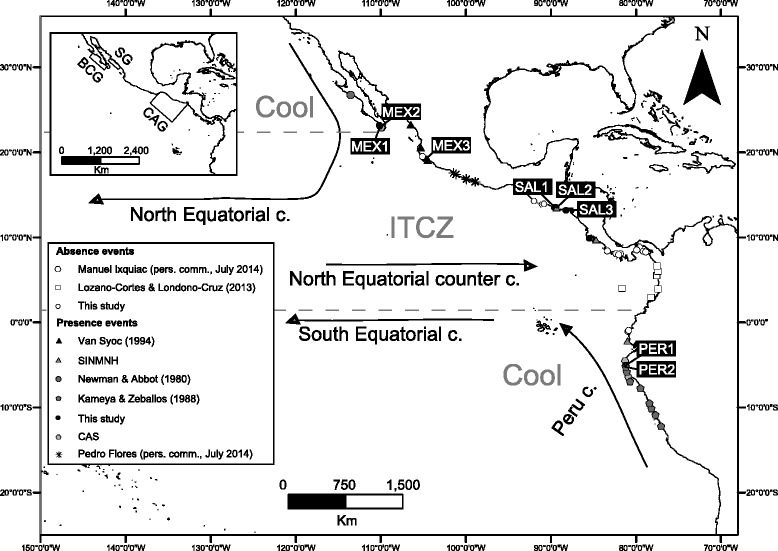
Map of the eastern tropical Pacific showing distribution of *Pollicipes elegans* and major surface current systems. Major current systems from [29]. ITCZ, Intertropical Convergence Zone. Presence and absence events as indicated in figure legend. Major regions without rocky intertidal habitat in the tropical eastern Pacific, the Sinaloan Gap (SG), the Central American Gap (CAG), and the mainland of Baja California Gap (BCG) from [41] are shown on the inset

The fragmented geographic distribution of the gooseneck barnacle, *Pollicipes elegans* [28], provides a potentially useful model to investigate the origin of a disjunct distribution in the tropical eastern Pacific (TEP). Extra-tropical populations of this single nominal species are abundant in north and central Mexico (MEX) and northern Peru (PER), areas that lie outside the Intertropical Convergence Zone (ITCZ), the warmest waters of the eastern Pacific. In contrast, records are rare in the ITCZ, between central MEX and northern PER (Fig. 1). Within the ITCZ (located predominantly to the north of the equator [29]), *P. elegans* is only known from El Salvador (SAL) and Costa Rica (CR) (Fig. 1), but few observations of the species are known from the two intervening areas to the north and south of SAL and CR [30–33]. Although the unusual distribution of *P. elegans* in the TEP has been referred to as antitropical [31, 33] or absent from the tropics, our more recent surveys of the region and the literature (Fig. 1) indicate that the fragmented distribution of *P. elegans* is best described as parantitropical [2], a trans-tropical distribution in which a species is more abundant towards the periphery than the center of its latitudinal range [30, 32].

The disjunctions in the geographic range of *P. elegans* could have been formed by either dispersal or vicariance. In a dispersal scenario, populations of *P. elegans* crossed the tropics in a stepping-stone fashion, from one hemisphere to the other, likely during periods of cooler climate [1, 2, 5, 34]. Alternatively, populations of *P. elegans* at the center of the species’ range could be relicts of a larger, continuous ancestral distribution. Under this scenario, the disjunct distribution arose primarily from decline of populations in tropical regions (presumably during warm interglacial climates) and the persistence of extra-tropical populations at the edge of the distribution.

Although *P. elegans* has high dispersal potential because their larvae spend 25–35 days in the plankton (S. Crickenberger, personal communication), mtDNA sequences (cytochrome oxidase I) gathered from extra-tropical populations (MEX and PER) showed a large sequence divergence (1.2 %) and near-reciprocal monophyly [33], suggesting limited or no genetic exchange across the TEP. Prior to the discovery of populations in El Salvador, Van Syoc [33] proposed that temperature was the primary factor limiting the colonization and persistence of populations in the warmest water of the TEP, and therefore gene flow across the TEP. Consistent with this idea, Walther et al. [35] found that larval thermal tolerance (measured by LT_50_, swimming performance, and respiration) of extra-tropical populations of *P. elegans* is lower than for larvae from tropical populations, and suggested a potential physiological barrier to gene flow into the tropics. In addition to temperature, habitat availability may also be an important factor influencing the distribution of *P. elegans*, given that the species only lives on rocky intertidal substrate with moderate to extreme wave action [30, 31]. The lack of such habitat over large stretches of the TEP coastline is well known [36–40]. The Sinaloan Gap (SG), the Central American gap (CAG), and the mainland Mexican coast of the Baja California GAP (BCG) (Fig. 1), are all areas dominated by sandy beaches and estuaries interrupted by only short stretches of rocky coastline [41, 42]. However, suitable habitat can be found north of the equator (in Ecuador, Colombia, and Panama), a region where *P. elegans* is absent, suggesting the disjunct distribution of *P. elegans* may be driven largely by temperature or potentially by a combination of factors in different regions (Fig. 1).

To build on Van Syoc’s [33] characterization of genetic diversity between extra-tropical populations in *P. elegans*, we used new (and larger) samples from all three regions of the species’ range (including previously unsampled SAL) and a statistical phylogeographic framework to evaluate models for the origins of the disjunct distribution of *P. elegans.* First, we gathered mtDNA sequence data that included tropical populations at the centre of the species' distribution to describe the range-wide population structure of this species. Then, using a combination of phylogenetic analyses, coalescent population genetic methods, and Approximate Bayesian Computation (ABC) we compared alternative biogeographical histories that varied in population separation order, relative isolation time, and ancestral N_e_, based on expectations of either trans-tropical dispersal or out-of-the tropics origin of extra-tropical populations models. If the disjunct distribution is a consequence of recent trans-tropical, stepping-stone dispersal, we expect an overall genetic signature of declining genetic diversity (e.g. [43]) from one hemisphere to the other and a match between summary statistics from the empirical data and those from simulations of stepping stone dispersal across the tropics. Alternatively, if the fragmented population is primarily a consequence of the loss of some tropical populations, we expect that genetic diversity should be either uniform across all regions or greatest in the tropics, and that summary statistics generated with out-of-the tropics origin simulations should match those observed in *P. elegans*. Although the biogeographical history of *P. elegans* may include both aspects of vicariance and dispersal, we show that genetic data can be used to distinguish an out-of-the tropics origin from a trans-tropical history of colonization.

## Results

### Sequence diversity statistics

After alignment, visual inspection, and trimming, a single 590 bp partial mitochondrial cytochrome c oxidase subunit I gene (COI) sequence was obtained for all individuals. Nucleotide diversity was greatest in the central (tropical) portion of the species range, where populations of *P. elegans* are currently most scarce; diversity peaked in SAL, with lower values in MEX and in PER (Table 1). Contrastingly, haplotype diversity was high in all sites, with a lower diversity at PER when compared to MEX and SAL.

**Table 1:** Molecular diversity and neutrality tests at the cytochrome c oxidase mitochondrial gene in populations of *Pollicipes elegans*

Population	Location	*n*	Hap	*π* ± *SD*	*H* ± *SD*	*k* ± *SD*	S	*R*_*2*_ (*P*)	Tajima’s *D* (*P*)	Fu’s *F*_*S*_ (*P*)
MEX1	Gaspareño, Mexico	25	21	0.006 ± 0.004	0.983 ± 0.017	3.713 ± 1.958	27	**0.0754** (0.036)	**-1.784** (0.016)	**-18.585** (0.000)
MEX2	Migriño, Mexico	21	15	0.005 ± 0.003	0.938 ± 0.040	2.924 ± 1.611	25	**0.0753** (0.013)	**-2.217** (0.006)	**-10.107** (0.000)
MEX3	Melaque, Mexico	24	21	0.008 ± 0.004	0.978 ± 0.024	4.486 ± 2.311	37	**0.0396** (0.000)	**-2.091** (0.007)	**-17.272** (0.000)
SAL1	Mizata, El Salvador	25	19	0.010 ± 0.006	0.970 ± 0.022	5.880 ± 2.940	30	0.0827 (0.080)	-0.972 (0.151)	**-9.086** (0.000)
SAL2	Taquillo, El Salvador	15	13	0.011 ± 0.006	0.981 ± 0.031	6.400 ± 3.249	24	0.1062 (0.116)	-0.552 (0.296)	**-5.275** (0.012)
SAL3	Jucuarán, El Salvador	31	19	0.008 ± 0.005	0.942 ± 0.025	4.976 ± 2.513	29	0.0733 (0.061)	-1.127 (0.135)	**-7.804** (0.003)
PER1	El Arco, Peru	20	14	0.007 ± 0.004	0.889 ± 0.068	4.237 ± 2.217	22	**0.0811** (0.026)	-1.214 (0.890)	**-6.080** (0.003)
PER2	Islilla, Peru	27	13	0.006 ± 0.004	0.889 ± 0.041	3.527 ± 1.870	16	0.1012 (0.274)	-0.522 (0.343)	-3.840 (0.042)

*n*, number of diploid individuals; Hap, number of haplotypes, *π*, nucleotide diversity; *SD*, standard deviation; *H*, haplotype diversity; *k*, number of haplotypes; S, number of segregation sites; *R*_*2*_, Ramos-Onsins and Rozas index; Tajima’s *D*; Fu’s *F*_*S*_ statistic. Significant departures from the null model of neutrality or population growth are shown in boldface; (*P*), p-value associated to neutrality tests

Given high haplotype diversity but very low nucleotide diversity, all three sequence neutrality statistics (Tajima’s *D*, Fu’s *F*_*S*_ and *R*_*2*_) were significant for all MEX populations, providing evidence of either a recent demographic expansion or a selective sweep in this region (Table 1). Overall, Fu’s *F*_*S*_ was significant in most of SAL and PER, but Tajima’s *D* and *R*_*2*_ were not. The mismatch distribution reflected the summary statistics from neutrality tests in that MEX populations presented a bimodal distribution, with one large peak relatively close to zero (mean =2.67) created by many sequences with few pairwise differences, plus a smaller peak due to the presence of a small number of more divergent haplotypes (See Additional file 1a-c). On average, *τ* was larger for PER (*τ* = 5.50, See Additional file 1 g-h) and SAL (*τ* = 6.09, See Additional file 1d-f) with SAL showing a mismatch distribution more ragged. The large number of sequences with few pairwise differences found in PER when compared with MEX could suggest a recent demographic expansion (See Additional file 1). Despite these differences, the sum of squared deviations and Harpending’s raggedness statistic were not significant for any population, indicating that a sudden demographic expansion could not be rejected for any sample (See Additional file 1).

### Phylogenetic analyses and population structure

**Fig. 2:**
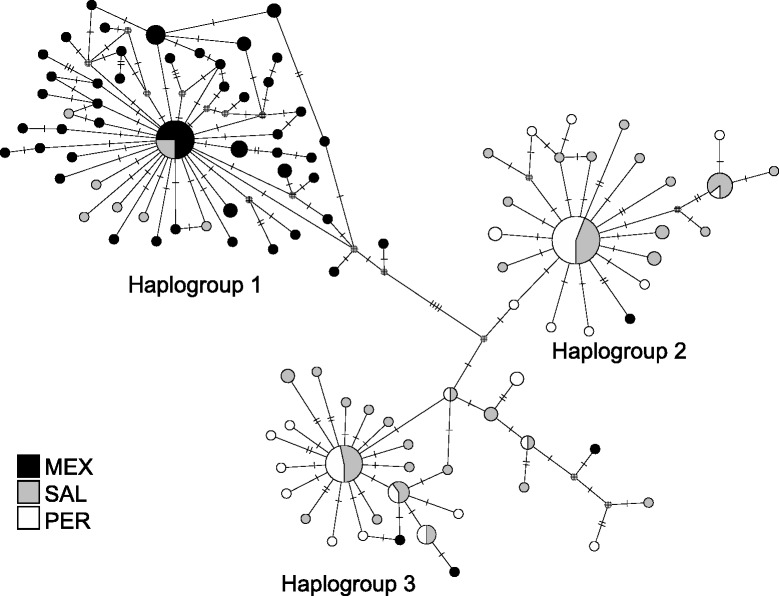
Unrooted gene network using the statistical parsimony algorithm with a confidence interval of 95 % based on cytochrome c oxidase sequences in *Pollicipes elegans* populations. Area of circles is proportional to haplotype frequency; smallest circle represent one haplotype. Sample origin is color coded as indicated in the figure legend. Circles with fill pattern represent intermediate, missing haplotypes. Perpendicular lines separating symbols represent number of mutational steps between haplotypes

**Fig. 3:**
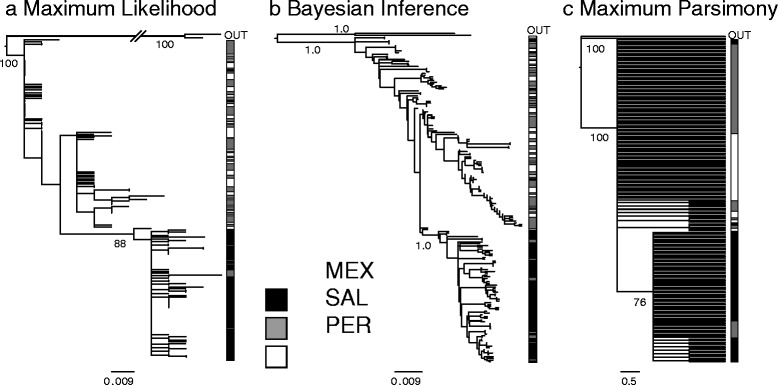
Phylogenetic inference based on cytochrome c oxidase sequences in *Pollicipes elegans* populations **a**. Maximum likelihood tree; **b**. Maximum credibility clade tree obtained with Bayesian inference; **c**. Maximum Parsimony tree. Tree inference was generated from partial sequences of the mitochondrial cytochrome c oxidase subunit I gene (COI) nucleotide sequence. Confidence support above the main nodes correspond to posterior probability for the Bayesian inference and bootstrap support for the likelihood and Parsimony inference. OUT represents sequences of *P. pollicipes* and *P. caboverdensis* used as outgroups in the analyses. Sample origin is color coded as indicated in the figure legend

The unrooted median joining haplotype network for COI had three main haplogroups and showed an association between haplotypes and geography (Fig. 2). Haplogroup 1 consisted primarily of individuals collected in MEX plus nine individuals from SAL. In contrast, haplogroups 2 and 3 primarily consisted of individuals from SAL and PER, with only four individuals from MEX (Fig. 2); both haplogroups 2 and 3 were well represented at SAL and PER sites. PartitionFinder found the best partitioning scheme with 1^st^, 2^nd^, and 3^rd^ codon positions partitioned independently. The best-fit model under BIC for the first, second and third codon position were GTR [44], F81 [45], and SYM + G [46], respectively. The rooted phylogenetic trees all (MP, ML and BI) revealed a consistent pattern in which haplogroup 1 (primarily MEX individuals) formed a monophyletic clade with high node support from bootstrap percentages and posterior probabilities (Fig. 3). Convergence of the BI runs was assessed by low average standard deviation of the split (>0.001), lack of patterns in the plot of the generation versus log probability, efficiency of Metropolis-Coupling MCMC sampler with acceptance rate for the cold chain higher than 10 % and lower than 70 %, and consistency among replicate analyses.

**Fig. 4:**
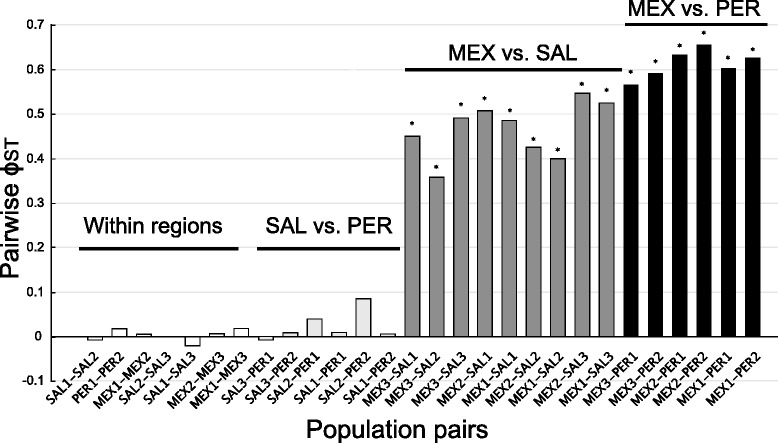
Pairwise Φ_ST_ among *Pollicipes elegans* populations. Population differentiation is presented from lower to higher geographical distance. Black bars represent population comparisons between Mexico and Peru populations. Grey bars represent comparisons between Mexico and El Salvador. Light grey bars correspond to El Salvador and Peru comparisons. White bars correspond to pairs of comparison between populations within the same region. Significant pairwise comparisons are shown with (*) after Bonferroni correction

Given the best-fitting model, we used the most similarly parameterized model implemented in Arlequin [47] to compare genetic diversity levels within sampling locations. The geographical patterns in the haplotype trees corresponded closely to very strong and significant genetic differentiation (Fig. 4) between MEX and each of PER (Φ_ST_ from 0.566 to 0.655) and SAL (Φ_ST_ from 0.358 to 0.507). In contrast, all pairwise Φ_ST_ estimates between SAL and PER were low and not significant (Fig. 4); estimates of Φ_ST_ between PER and SAL were similar to values between populations within each region (Fig. 4). The SAMOVA analysis reflected these patterns, maximizing the variance among groups relative to the total variance (F_CT_) when MEX populations were considered as one group and all SAL and PER populations as another group (Table 2).

**Table 2:** Fixation indices and percentage of variation explained by each source for groups of populations identified by SAMOVA within each clade (all results are significant at *P <* 0.05 and shown in boldface).F_SC_, variance among subpopulations within groups; F_ST_, variance among subpopulations relative to the total variance; F_CT_, variance among groups relative to the total variance

		Fixation indices			Percentage variation		
K	Group compositions^a^	F_SC_	F_ST_	F_CT_	Among groups	Among populations	Within populations
2	(MEX1, MEX2, MEX3) - (SAL1, SAL2, SAL3, PER1, PER2)	0.010	**0.526**	**0.522**	52.190	0.460	47.350
3	(MEX1, MEX2) - (MEX3) - (SAL1, SAL2, SAL3, PER1, PER2)	0.009	**0.495**	**0.491**	49.070	0.470	50.450
4	(MEX1) - (MEX2) - (MEX3) - (SAL1, SAL2, SAL3, PER1, PER2)	**0.013**	**0.481**	**0.475**	47.450	0.690	51.860
5	(MEX1) - (MEX2) - (MEX3) - (SAL2) - (SAL1, SAL3, PER1, PER2)	**0.002**	**0.447**	**0.446**	44.580	0.120	55.300
6	(MEX1) - (MEX2) - (MEX3) - (SAL2) - (PER1) - (SAL1, PER1, PER2)	-0.001	**0.414**	**0.414**	41.450	-0.040	58.590
7	(MEX1, MEX2) - (MEX3) - (SAL1) - (SAL2) - (SAL3) - (PER1) - (PER2)	-0.007	**0.384**	0.388	38.790	-0.400	61.610

^a^Population names as in Table 1

#### Skyride plots

**Fig. 5:**
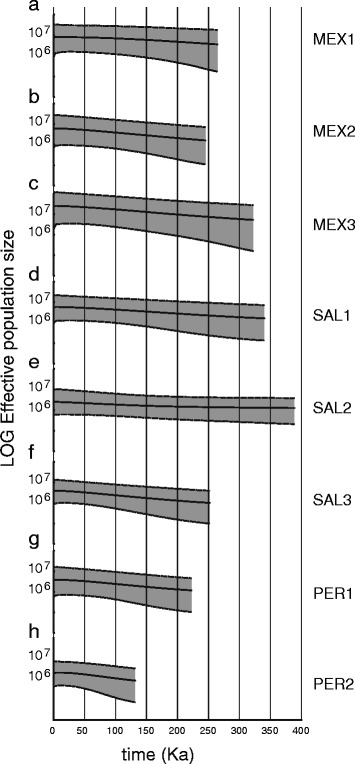
Bayesian skyride plots of the logarithm of N_e_ change through time. The black line indicates the mean posterior N_e_ through time. The gray area represents the 95 % HPDI, taking into account coalescent model and phylogenetic uncertainty. Panel **a**, **b** and **c** stand for MEX sites. Panel **d**, **e** and **f** stand for SAL sites. Panel **g** and **h** stand for PER sites

Using the best-fitting substitution model, we found that, like the sequence diversity statistics, Bayesian skyride plots lacked strong evidence of recent changes in N_e_ (Fig. 5), with demographic histories that did not differ significantly from a constant size model. However, the time to the most recent common mtDNA ancestor estimates were consistently older towards the centre of the range of *P. elegans*, with SAL2 being the oldest and PER2 the youngest site (Fig. 5).

### Coalescent-based demographic parameter estimates

Likelihood ratio tests from the isolation with migration (IM) analysis showed evidence for migration between *P. elegans* populations in only three of 28 pairwise comparisons. These three comparisons were not significant after a Bonferroni correction for multiple tests (See Additional file 2). In all 28 comparisons, the joint posterior density for m increased asymptotically as m approached zero, indicating either no gene flow across populations or a lack of migration signal in the data (See Additional file 3).

Divergence times varied among pairwise comparisons, with more old divergences among populations within MEX than compared to populations within SAL and PER (See Additional file 4). The posterior density distribution for the divergence times between MEX and PER (~150 to 300 ka) overlapped with the divergence times between MEX and SAL (~150 to 250 ka). However, the divergence time between SAL and PER showed consistently more recent and narrower joint posterior density distributions (~50 to 150 ka) (See Additional file 4), similar to divergence times among localities within SAL and PER.

Only a few posteriors for N_e_ showed a well-defined peak with tails that returned to zero (See Additional file 5). However, estimates of N_e_ towards the centre of the species’ range were potentially very large, especially when compared to PER populations. Considering only the posterior density plots with clear peaks, SAL showed only a slightly higher mean historical N_e_ (2 x 10^6^ individuals) than either MEX (1.5 x 10^6^ individuals) or PER (7.5 x 10^5^ individuals) (See Additional file 5).

### Approximate Bayesian computation phylogeography

**Fig. 6:**
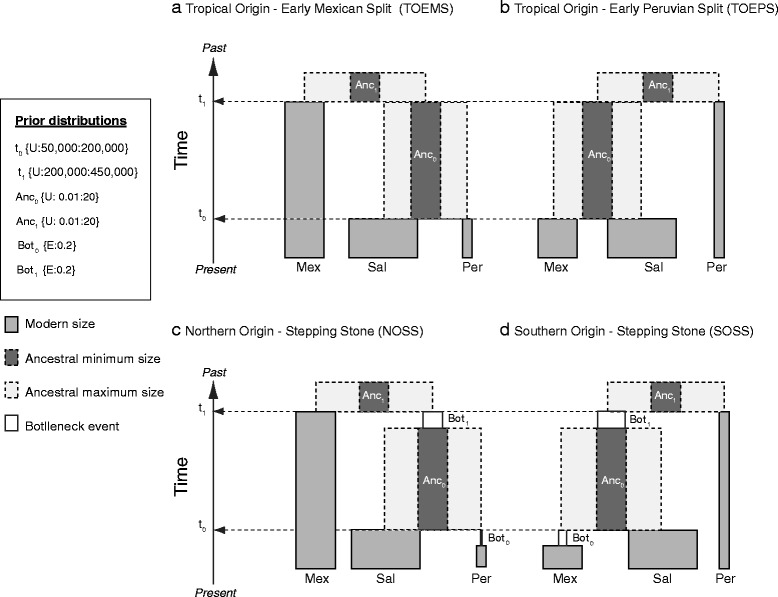
Representation of the demographic models simulated with BSSC followed by Approximate Bayesian Computation to evaluate the plausibility of vicariance and dispersal as the origin of parantitropical distribution of *Pollicipes elegans.* T_0–1_ time of splitting event; Anc_0–1_ Ancestral N_e_ at the moment of splitting; Bot _0–1_ bottleneck events before splitting events. Panel **a** and **b** represent vicariance demographic models with a tropical origin. Panel **c** and **d** represent stepping stone dispersal demographic models

Simultaneous evaluation of all four competing models indicated that Tropical Origin, Early Mexican Split (TOEMS, Fig. 6a) had the highest posterior probability (PP) value (average PP = 0.74), indicating that TOEMS was the best-fitting model for all tolerance levels. When comparing all models simultaneously, cross validation averaged 67 % (Table 3). However, in the pairwise model comparisons, cross validation support improved substantially (mean = 93 %), and as in the simultaneous evaluation of models, TOEMS was consistently selected as the best fit model, with the highest posterior probability value (average PP = 0.89) (pairwise results not shown).

**Table 3:** Cross validation and multiple phylogeographic model selection results under feed forward neural network method

Model	δ	Samples	Cross validation (%)	Posterior probability
NOSS	0.001	4,000	83	0.2433
	0.002	8,000	84	0.2694
	0.005	20,000	86	0.1183
	0.01	40,000	84	0.3667
SOSS	0.001	4,000	93	0.0020
	0.002	8,000	97	0.0065
	0.005	20,000	96	0.0033
	0.01	40,000	95	0.0332
TOEMS	0.001	4,000	61	0.7543
	0.002	8,000	69	0.7234
	0.005	20,000	73	0.8781
	0.01	40,000	65	0.5966
TOEPS	0.001	4,000	79	0.0003
	0.002	8,000	84	0.0007
	0.005	20,000	78	0.0004
	0.01	40,000	72	0.0035

δ, tolerance level

Estimates of ancestral effective population size (Anc_0_ and Anc_1_) and time of divergence (t_0_ and t_1_) for the best-fitting model (TOEMS) showed contrasting patterns between the northern and southern portions of the species’ range. In the north, MEX and SAL separated first (330 ka ago, Table 4, t_1_, δ = 0.001), from a common ancestor that was 35 % of the historical N_e_ of SAL populations found with IMa2 (Table 4, Anc_1_, δ = 0.001). However, in the south, PER separated from SAL far more recently (56 ka ago, Table 4, t_0_, δ = 0.001) from a common ancestor in the tropics that was much smaller, only 3 % of the averaged historical N_e_ over the coalescent of SAL populations (Table 4, Anc_0_, δ = 0.001). Overall, diagnostic plots of parameters Anc and t suggested little influence of the tolerance level and priors in the posterior density plots (Table 4) and a cross validation of the estimated parameters showed a minimum effect of the tolerance level in the error rate.

**Table 4:** Parameter estimates in the approximate Bayesian computation (ABC) analysis of Tropical Origin, Early Mexican Split (TOEMS) model. Parameter estimates correspond to the weighted median, mean and mode of the posterior samples

δ	Samples	t_0_	Anc_0_	t_1_	Anc_1_
0.001	1000	60,345 / 61,202 / 58,979	0.0426 / 0.0520 / 0.0321	336,399 / 330,799 / 360,096	0.3163 / 0.3555 / 0.1562
0.002	2000	77,074 / 78,702 / 74,209	0.0480 / 0.0558 / 0.0375	326,858 / 319,446 / 348,733	0.1767 / 0.1984 / 0.0995
0.005	5000	68,409 / 69,455 / 68,666	0.0433 / 0.0540 / 0.0329	315,571 / 308,276 / 365,371	3.8392 / 4.0773 / 2.8303
0.01	10000	65,698 / 66,783 / 62,755	0.0932 / 0.2298 / 0.0620	315,799 / 308,359 / 362,294	4.5673 / 4.9697 / 1.5832

δ, tolerance level; t_0_, Anc_0_, t_1_, Anc_1_ as described on the methods and in Figure 6

## Discussion

Spatial patterns of genetic variation in *Pollicipes elegans* did not support a scenario of recent trans-tropical colonization but instead were most consistent with a tropical origin hypothesis. First, a peak in nucleotide diversity in SAL (tropical region) is not consistent with recent dispersal across the tropics: in a trans-tropical dispersal scenario, sequential colonization events should leave behind a signature of declining genetic diversity from north to south or from south to north, similar to what has been observed in many other taxa that have recently expanded their geographical range in one direction over time (e.g. [48, 49]). Although haplotype diversity did not peak in the tropics, high haplotype diversity in MEX appears to be the result of a demographic expansion, not a consequence of a origin from a tropical ancestor. Viewed from the perspective of the coalescent, Bayesian skyride plots showed that haplotypes in tropical populations had a consistently deeper history compared to haplotypes in all other populations to the north or south, a pattern recovered in other coastal and terrestrial tropical species, often associated with distribution changes as consequence of Quaternary glaciations, indicating either a relatively larger historical N_e_ or older population ages in the tropical region [50].

Although the overall patterns of genetic diversity found in *P. elegans* supports a scenario involving a more diverse and possibly older and/or larger SAL populations, we used ABC model comparison to investigate which history fits best to the observed data. Our ABC comparisons found that a tropical origin model was a better fit for the data than a trans-tropical dispersal model. Under the best-fitting tropical origin scenario, *P. elegans* was abundant and widely distributed throughout the tropics, but through the loss of most populations in the tropical region (perhaps coupled with colonization at higher latitudes), the species’ range was sequentially divided into three isolated populations. Differences in estimates of historical N_e_ across regions and over time detected with ABC also revealed a complex history of changes in population size with respect to population separations. For example, instead of reflecting recent colonization of PER from SAL, the relatively low mtDNA diversity in PER when compared to MEX and SAL, reflects the relatively small current population size of PER and the relatively long period of time that the ancestral population (Anc_0_ from t_1_ to t_0_) spent at a relatively small population size, potentially reflecting the predominance of cold glacial periods over the last 400 ka. In contrast, the high mtDNA diversity of the SAL populations can be attributed to the relatively recent large increase in population size that happened after the separation of PER at t_0_, a result that is not evident from the mismatch or skyride analyses. However, skyride plots also showed no evidence of recent variation in N_e_ over time in PER, also consistent with an extinction model rather than a history punctuated by a recent colonization events (See Additional file 1i-p).

Even though our model comparison using ABC strongly favored a tropical origin model over a trans-equatorial stepping-stone model, we recognize that our inferences are based on summary statistics from a single mtDNA gene. Deep phylogeographic incongruences between mtDNA and multilocus nuclear data are rare [51], but some of the unexpected details that emerged from our mtDNA study warrant acquisition of additional data (i.e., more loci). For example, although IM methods showed little evidence of any gene flow among the three disjunct regions of Mexico, El Salvador, and Peru, these methods also failed to find much evidence of gene flow within regions on smaller spatial scales. Because some crustacean larvae, including barnacles, are able to regulate depth and may be locally retained or returned to coastal habitats after initial transport offshore [52–55], we cannot ignore the potential for nearshore larval retention in *P. elegans*. At the same time, however, we realize that although mtDNA is likely to retain information about major biogeographic events, estimates of gene flow could be downwardly biased by the exclusive use of mtDNA, which may rapidly lose information about gene flow given the smaller effective population size of mtDNA relative to nuclear loci. The use of additional independent nuclear markers in the future may validate our inferences about the demographic history of *P. elegans*.

### **Modern and historical factors shaping the distribution of***Pollicipes elegans*

Several historical and contemporary factors can potentially explain the formation of the disjunct distribution and the persistence of apparently relict tropical populations in the warmest region of the TEP. First, the current range disjunction and restricted connectivity between tropical and extra-tropical populations could be influenced by the geographical distribution of suitable adult habitat. The tropical population at the center of the range of *P. elegans* lies in the Central American Gap or CAG [36, 41], a transition zone between the Mexican and Panamanian Zoogeographic Provinces [31, 56] that is dominated by sandy or muddy coastline interspersed with mangrove-lined lagoons [36–40]. Although our data cannot directly address this ecological issue, the absence of suitable habitats for *P. elegans* across large stretches of this region may substantially limit the distribution of *P. elegans*, especially along the northern CAG coastline between existing SAL and MEX populations, where rocky shores are far apart from each other and may provide a total barrier to gene flow (Fig. 1). However, rocky shore habitat is common south of the CAG where *P. elegans* is absent over 2,000 km of shore from SAL to northern Ecuador (Fig. 1), suggesting that other factors may prevent the colonization and persistence of *P. elegans* in that region.

A second explanation for the formation and maintenance of the disjunct distribution of *P. elegans* could be explained by the presence of a physical and/or physiological barrier to larval dispersal. The clockwise rotation of the north Pacific subtropical gyre and the counterclockwise rotation of the south Pacific gyre create a convergence of surface waters in the warmest area of the TEP (Fig. 1; for a review see [29]). This circulation pattern pushes water away from the coast at the northern and southern margins of the ITCZ (Fig. 1), which could limit larval dispersal both in and out of the ITCZ. Larvae from extra-tropical regions could potentially move all the way across the tropical Pacific in the northern and southern equatorial currents (Fig. 1) and then be returned back to the TEP via the northern equatorial counter current [5], but Walther et al. [35] showed that the high temperatures in the ITCZ likely present a significant physiological barrier to dispersal of larvae from cooler regions. Although temperature effects on larvae may explain why there is little (if any) gene flow into the ITCZ, it does not, however, explain why gene flow out of the ITCZ appears to be similarly restricted in *P. elegans*. Recruitment in the CAG could be limited by juvenile and adult survival; adult temperature tolerances have yet to be investigated in this species. Comparable patterns of geographical differentiation have been found in the barnacle *Chathamalus* [57] and the sand crab *Emerita analoga* [58], with divergence of northern and southern clades similar to the one found in *P. elegans*, around 400 ka and 250 ka respectively. Other marine species with genetic structuring within the ITCZ include oysters, seahorses, and corals [59–61].

Although modern environmental factors (e.g. sea surface temperature and habitat availability) may currently regulate the distribution of *P. elegans*, historical environmental changes may have caused the initial range disruption. Large fluctuations in sea level, patterns of ocean circulation, and sea surface temperature have been invoked as important factors contributing to diversification of marine species in the TEP [62, 63], but see [64]. At the outset of our study, we expected that if a tropical origin scenario best explained the disjunct distribution of *P. elegans*, population separation times should correspond to relatively warm interglacial climates when the majority of tropical populations were extinct. However, acknowledging the uncertainty in mutation rates [65–67], the use of a single genetic marker, and that parameter estimates based on summary statistics may be sensitive to the choice of summary statistics used in ABC [68–70], both the IM and ABC methods indicated the unexpected pattern that isolation of both extra-tropical populations may have occurred during cold glacial periods, suggesting that MEX and PER populations became extra-tropical isolates when the main range of the species likely contracted towards the tropics. Strong advection of cold water from the Peruvian Current system during glacial cycles may have also generated a latitudinal shift of the Intertropical Convergence Zone northward, disrupting gene flow in the equatorial region by sudden contraction of populations north of the equator [71]. The intensification of upwelling in the eastern equatorial ocean during the mid-Pleistocene climate transition, followed by an increase in temperature in the equatorial Pacific Ocean, might also have strongly affected connectivity across the TEP [72–74].

The effects of decadal variation in temperature on connectivity and persistence of modern marine populations may also have left an imprint on population structure and regional patterns of genetic diversity in *P. elegans*. El Niño Southern Oscillation (ENSO) and acute seasonal variation have profoundly impacted population and community dynamics through repeated regional mortality events and extra-tropical colonization events, especially in South America [75]. For example, during the El Niño event of 1997–1998, high sea surface temperature and run-off of terrestrial guano during intense rainfall seasons caused mass mortalities of *P. elegans* in Lobos de Afuera island, a location 200 km away from the PER2 site of this study [76]; during the 1982–1983 El Niño, *P. elegans* colonized rocky shores of central Peru, establishing a local short-lived local fishery [77, 78]. Our data show that N_e_ among PER populations has been considerably smaller than either SAL or MEX (See Additional file 5), which, in addition to having been derived from a relatively small ancestral population, may also reflect repeated bottleneck events caused by extinction and colonization in Peru during strong ENSO years, rather than consistently small N_e_.

### Evolutionarily significant units within *Pollicipes elegans*

Speciation in marine taxa like *P. elegans* with high dispersal potential remains incompletely understood [6, 79–81]. The very strong phylogeographic break (i.e., near reciprocal monophyly of mtDNA) between MEX and SAL/PER haplogroups suggest that MEX populations could be on an independent evolutionary trajectory with respect to all populations to the south of the CAG. The strong phylogeographical differentiation that has persisted across the last two glacial-interglacial cycles (150–300 ka) indicates that two evolutionarily significant units may exist within *P. elegans*, which explain the differential demographic responses of Mexican populations as compared to all others to the south. Incomplete lineage sorting of mtDNA haplotypes (with little detectable gene flow) between PER and SAL populations suggest that PER populations could provide an earlier snapshot in the process of speciation between tropical and extra-tropical populations. With near reciprocal monophyly, a future study with multi-locus species delimitation methods would be appropriate to address the presence of cryptic species within *P. elegans.*

## Conclusion

Although taxa with disjunct distributions have been the subject of intensive biogeographic research [82–85], comparisons of alternative hypotheses of historical divergence based on modeling of demographic history are still rare. Using a combination of coalescent population genetic analyses and ABC modeling from data collected from a single mtDNA gene, our study found very little support for a history of trans-tropical dispersal in the gooseneck barnacle *P. elegans*. Instead, our results support the idea that the extra-tropical populations of *P. elegans* likely developed through a process of population separations that was not dominated by dispersal across uninhabitable regions, but instead best described by a tropical origin model. Further, our data and analyses indicate that for *P. elegans*, the separation of extra-tropical populations from central populations in the warmest tropical water was achieved in two temporally distinct steps: the isolation of MEX populations followed by a more recent isolation of PER populations, both potentially during cold interglacial climates.

The formation of ephemeral dispersal barriers that arose during sea level fluctuations in the Pleistocene [62, 86, 87] combined with cycles of local extinction and recolonization, have been proposed as opportunities for rapid speciation of marine species in the tropics [6, 88]. Using frequency-based statistics, coalescent methods, and ABC modeling, our results consistently supported a tropical origin likely driven by vicariance, rather than dispersal across unsuitable habitat, as the main mechanism that produced the largely antitropical distribution of *P. elegans* in the eastern Pacific. Drivers of the modern disjunct geographical distribution and isolation of populations of *P. elegans* are likely explained by habitat availability, ocean circulation, and physiological response to thermal stress. Future studies can address if the distinct evolutionary significant units found in MEX and Southern MEX correspond to a novel example of cryptic speciation along a steep latitudinal thermal gradient and validate our inferences about the demographic history of *P. elegans* using additional independent nuclear markers.

## Methods

### Geographic sampling, DNA isolation and gene sequencing

Adult individuals of *Pollicipes elegans* were collected between 2009 and 2011 from sites chosen to capture variation in populations at the north, south, and centre of the species’ range (Fig. 1a). A total of 178 adult individuals were collected across three different sites in MEX and SAL, and from two places in PER (Fig. 1, Table 1). Tissue samples from the peduncle (muscle) of live barnacles were preserved in 70 % ethanol. Genomic DNA was extracted by overnight proteinase K incubation in 2X cetyl trimethyl ammonium bromide (CTAB) followed by two chloroform extractions and recovered with ethanol precipitation. LCO1490 and HCO2198 universal primers [89] were used to amplify a portion of COI. Polymerase chain reaction (PCR) was carried out in 20 μL volumes containing 1x PCR buffer, 3 mM MgCl_2_, 0.4 mM each dNTPs, 0.5 U of GoTaq DNA polymerase (Promega), 0.3 μM each primer, and approximately 50 ng of genomic DNA. Amplification conditions consisted of 94 °C for 2 min followed by 5 cycles of 94 °C for 30 s, 42 °C for 1:30 min, and 72 °C for 1 min. These first 5 cycles were followed by 35 cycles of 94 °C for 30 s, 49 °C for 1 min, 72 °C for 1 min, with a final incubation step at 72 °C for 5 min. PCR products were purified using QIAquick spin filter columns (QIAgen) and sequenced in both directions on an ABI-3730 sequencer (Applied Biosystems). The sequences were inspected for base-calling errors with the forward and reverse strands aligned using Sequencher 4.7 (Gene Codes Corporation). Sequences were edited using BioEdit [90] and easily aligned with ClustalW [91]. Sequences are available in GenBank at the NCBI [GenBank:KF958514-KF958701].

### Sequence diversity statistics, mismatch distribution and Skyride plots

Frequency based statistics were calculated to evaluate patterns of genetic diversity and demographic history across the distribution range of *P. elegans*. Nucleotide diversity (*π*), haplotype diversity (*H*), pairwise differences (*k*), segregating sites (*S*), Tajima’s *D* [92], and Fu’s *F*_*S*_ [93] were calculated with Arlequin version 3.5.1.2 [94] with 10,000 permutations to test significance. *R*_*2*_ [95] was calculated with DnaSP version 5 [96] with 10,000 coalescent simulations. To assess the demographic history at each sampling site, we calculated nucleotide mismatch distribution plots, the sum of squared deviations (*SSD*), Harpending’s *r*, and the parameter tau (*τ*) in Arlequin to evaluate the fit of a sudden demographic expansion. The historical demography at each site was reconstructed by using Bayesian Markov Chain (MCMC) method implemented in Beast version 1.7.2 [97]. The analysis was executed with a codon-structured substitution model found with PartitionFinder version 1.1.1 [98], a relaxed uncorrelated lognormal clock, and a Gaussian Markov Random Field (GMRF) coalescent prior [99]. Each GMRF skyride analysis was conducted twice for 10,000,000 generations each, sampling every 1,000 generations. We used the mean of trans-isthmian estimates of COI divergence rates from the most recently separated species among crabs, snapping shrimp and barnacles (1.32 %, 1.7 % and 3 % per My, respectively) [100–102] to calculate a rate of 1x10^−8^ substitutions per site per year and perform the analysis. Although *Chthamalus* barnacles are phylogenetically closer to *Pollicipes* (~239 Ma) [103], we included additional geminate-pair rates in our calculations because *Chthamalus* significantly faster rate could reflect a separation that started earlier than the closure of the panama isthmus, resulting in overestimates of the evolution rate. Therefore, using an average rate across crustacean taxa (i.e. 2 % per My) is a conservative approach and appropriate for this study. The output files were checked in Tracer version 1.5 to ensure that all effective sampling sizes (ESS) values were greater than 200. We considered population size changes as significant when the upper and lower 95 % confidence intervals at the root and the tips did not overlap.

### Phylogenetic analyses and population structure

Phylogenetic relationships were inferred using Bayesian Inference (BI), Maximum Likelihood (ML), and Maximum Parsimony (MP). For the model-based methods, the best sequence partition strategy and best-fitting model of nucleotide substitution was identified with PartitionFinder using the Bayesian Information Criterion (BIC). BI analyses were performed using MrBayes version 3.1.2 [104], with five independent runs of four Markov chains for 10 million generations and default heating values, sampling every 100 generations with 2500 samples discarded as burn-in. A maximum credibility clade tree was generated using TreeAnnotator version 1.8.2 [97] from a combined distribution of topologies with a posterior probability threshold of 0.5 and median node heights. ML analyses were conducted using RAxML version 7.04 [105]. Nodal support was estimated from 1,000 fast bootstrap replicates in five independent runs. MP was implemented using PAUP* 4.0b10a [106], with heuristic searches, tree bisection reconnection, and with 1,000 random addition replicates with all characters treated as unordered and equally weighted. Node reliabilities in the MP tree were assessed with 1,000 bootstrap replicates and averaged over five independent runs. Additionally, an unrooted gene network using the statistical parsimony algorithm was built using a confidence interval of 95 % in TCS [107]. Partial COI sequences from *P. pollicipes* (GenBank:HM563669) and *P. caboverdensis* (GenBank:HM563667) were retrieved from GenBank database and used as outgroups.

Genetic structure was characterized with Φ_ST_, a measure of the proportion of nucleotide diversity within sub-populations relative to the total. We used the substitution model in Arlequin most similar to the model identified with PartitionFinder. Significance was assessed with 10,000 permutations of the data and a Bonferroni correction was used to reduce the chances of obtaining false positives with multiple pairwise comparisons. A Spatial Analysis of Molecular Variance (SAMOVA) was then used to define groups of populations by maximizing the proportion of total genetic variance due to differences between groups of populations in SAMOVA version 1.0 [108].

### Demographic parameter estimates using Isolation-with-migration model

We estimated demographic parameters by sampling the coalescent in a Bayesian framework with the program IMa2 [109], which is based on an isolation-with-migration (IM) model [109–111] that uses Metropolis-coupled Markov-chain techniques to estimate the posterior densities of the time of divergence (t), theta (Θ) and migration (m). To obtain parameter estimates in demographic units (i.e. time in years, effective population size and number of migrants per generation as number of individuals), the length of the COI gene sequences and a generation time of one year, previously described for the congeneric species *P. pollicipes* [112, 113] was considered to calculate a substitution rate of 5.9 x 10^−6^ mutations per locus per generation. Initial 8 h IMa2 runs (with a burn-in of 8 h) of forty independent heated chains [114] were performed to assess if the priors were suitable and if the heating conditions were appropriate. We then conducted two longer independent runs (100,005 saved genealogies) for each analysis. Stationarity was assessed by comparisons between parameter estimates generated from genealogies from the first and the second half of the run, and by visual inspection of the splitting time trend plots [109].

Likelihood ratio tests (LRTs) were computed to test the significance of migration (m = 0) between each pair of populations [109, 110]. Because the LRT statistic is not a good fit to the theoretical expectation until higher values of LRT are achieved, we used the same approach as [115], by considering LRT values less than 1 as a failure to reject the hypothesis of m = 0 ; values greater than 1 but lower than 2.74 viewed as inconclusive [109]; values greater than 2.74 were considered significant. A Bonferroni correction was used to reduce false positives (type I errors) with multiple pairwise comparisons.

### Approximate Bayesian computation

To comprehensively evaluate the likelihood of different demographic scenarios, we used an Approximate Bayesian Computation (ABC) approach [116]. Bayesian Serial Simcoal (BSSC) [117] was used to determine if the observed nucleotide and haplotype diversity, number of segregating sites, genetic differentiation F_ST_ and Tajima’s D could be explained as a result of one of four specific demographic models (Fig. 6). Given that there was no significant genetic differentiation within any of MEX, SAL, or PER (see Results) we pooled samples within regions (peripheral and centre) to reduce the number of scenarios and parameters to be analyzed. We evaluated four basic biogeographic models of historical divergence: a) Tropical Origin, Early Mexican Split (TOEMS) (Fig. 6a), a scenario that proposes MEX populations split away from an ancestral central (tropical) population today represented by the population in SAL, followed by a split between SAL and PER; b) Tropical Origin, Early Peruvian Split (TOEPS) (Fig. 6b), a history in which PER diverged from a tropical SAL ancestral population before MEX populations diverged from SAL; c) Northern Origin Stepping-Stone (NOSS), and d) Southern Origin Stepping-Stone (SOSS), models consistent with colonization from one hemisphere to the other (Fig. 6c-d).

In all models, an average of parameter estimates obtained from IMa2 runs were used to define the prior boundaries in Bayesian simulation scenarios (Fig. 6). The split (disruption of connectivity) between MEX and SAL, and MEX and PER populations could be produced either by dispersal into un-colonized regions (Fig. 6c-d) in time t_0_ and t_1_, or by extinction of intermediate populations (vicariance) (Fig. 6a-b). The models differ with respect to the order of population splits, the geographic location of ancestral populations, and changes in population size. Models a and b both assume that the El Salvador population is the oldest population in the region and that Mexico (Fig. 6a) and Peru (Fig. 6b) were sequentially separated from this central and ancestral population in El Salvador. In contrast, Models c and d assume that the oldest population is at one end of the modern distribution of *P. elegans,* represented today by either Mexico (Fig. 6c) or Peru (Fig. 6d) and that two successive colonization (sampling) events created the modern distribution. Each colonization was modeled by a bottleneck on population size (parameters Bot_0_ and Bot_1_) in the colonized region immediately after the split of populations. Each bottleneck event was assigned a prior exponential distribution centered in 20 % of the modern N_e_. The sizes of ancestral populations after population splits were represented by parameters Anc_0_ and Anc_1._ This parameter is obtained by modeling the ancestral population size at t_0_ and t_1_ to be any number between one and twenty times the size of the modern N_e_.

Each scenario was tested by modeling 1,000,000 coalescent simulations (4 million in total). We conducted model fitting in R using abc package [118] with feed-forward neural networks (nonlinear regression). Neural networks reduce a large number of summary statistics into a smaller number of dimensions [119], a more robust and accurate approach when the number of summary statistics is large [120]. Cross validation was performed with four tolerance levels (δ = 0.001, 0.002, 0.005, and 0.01) and 100 steps. Competing models were compared based on their posterior probability and Bayes Factor scores. To ensure that the model selection procedure was reliable, model selection and cross-validation steps were performed twice, first by comparing all four models against each other simultaneously and then by pairwise comparisons of all models. Parameter estimates for splitting times (parameters t_0_ and t_1_) and ancestral N_e_ (parameters Anc_0_ and Anc_1_) were estimated for the best-fitting model and validated using four tolerance levels.

## Additional files

Additional file 1:
**Mismatch distributions for each sampling site.** Mismatch distributions for each sampling site of *Pollicipes elegans.* Bars represent observed values and lines indicate the mismatch distribution expected from a sudden expansion model. SSD and HRI correspond to sum of squared deviations and Harpending’s ruggedness index respectively. (PDF 210 kb) Additional file 2:
**Migration rate parameter estimates.** Likelihood ratio test (LLRtest) for migration rate parameters *m*, and population migration *2NM* between pairs of populations of *P. elegans*. (DOCX 84 kb) Additional file 3:
**Migration rate posterior plots.** Joint posterior density plots of parameter m between populations obtained from IMa2 analyses based on data from COI locus of *Pollicipes elegans*. (PDF 1062 kb) Additional file 4:
**Divergence time posterior plots.** Joint posterior density plots of divergence time between populations obtained from IMa2 analyses based on data from COI locus of *Pollicipes elegans*. (PDF 3496 kb) Additional file 5:
**Population size posterior plots.** Joint posterior density plots of number of individuals obtained from IMa2 analyses based on data from COI locus of *Pollicipes elegans*. (PDF 4467 kb)
