# Supplementary figures and images for: Out-of-the tropics or trans-tropical dispersal? The origins of the disjunct distribution of the gooseneck barnacle Pollicipes elegans
Source: Front Zool. 2015 Dec 30;12:39. doi: 10.1186/s12983-015-0131-z (PMC4696079; doi:10.1186/s12983-015-0131-z)

Frequency

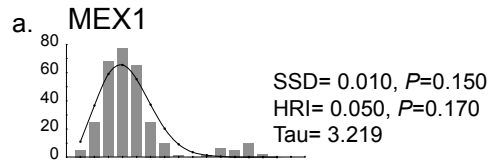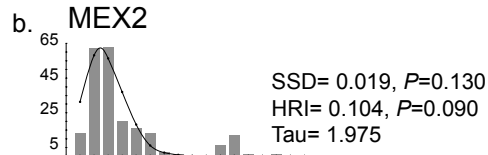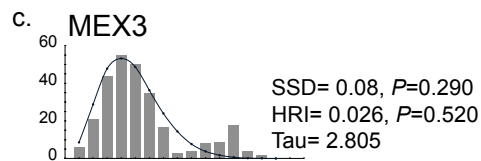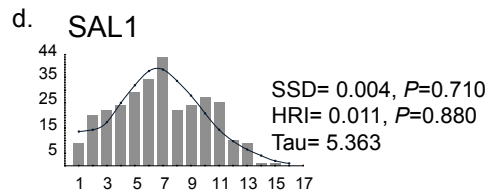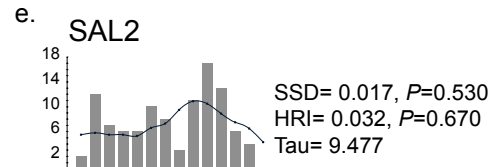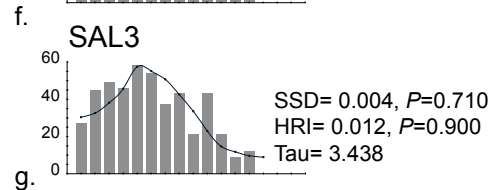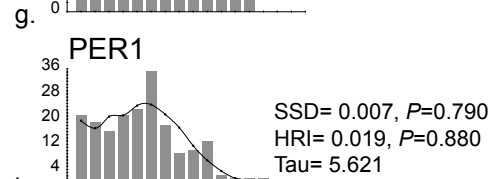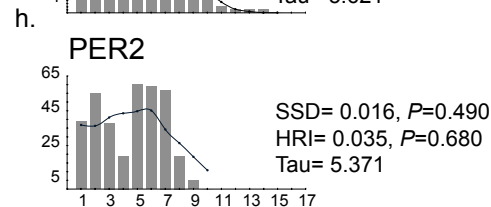

Number of Differences

Supplement: Additional file 1: — Mismatch distributions for each sampling site. Mismatch distributions for each sampling site of Pollicipes elegans. Bars represent observed values and lines indicate the mismatch distribution expected from a sudden expansion model. SSD and HRI correspond to sum of squared deviations and Harpending’s ruggedness index respectively. (PDF 210 kb) [file 12983_2015_131_MOESM1_ESM.pdf]

$P(m/X)$

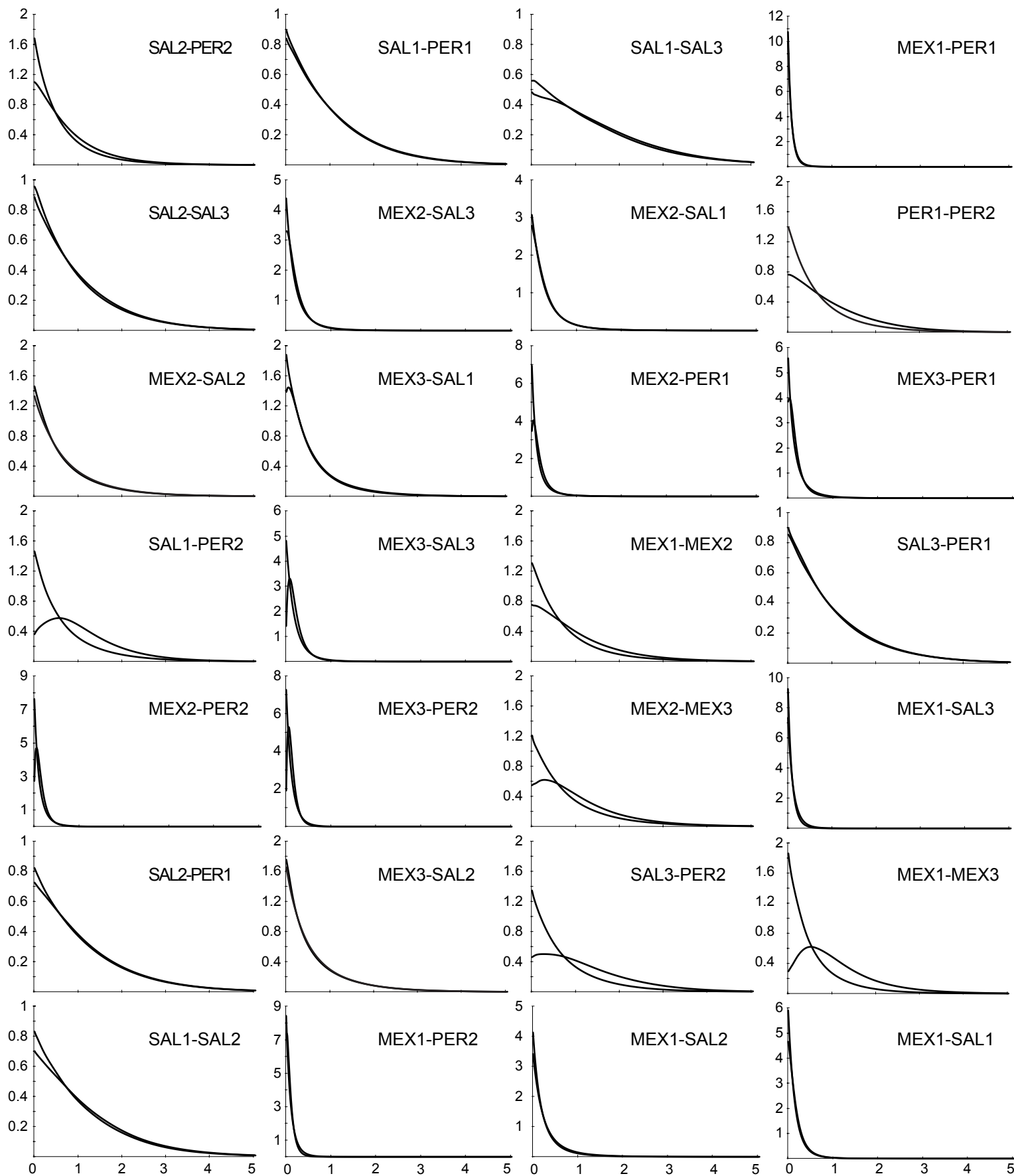

Migration

Supplement: Additional file 3: — Migration rate posterior plots. Joint posterior density plots of parameter m between populations obtained from IMa2 analyses based on data from COI locus of Pollicipes elegans. (PDF 1062 kb) [file 12983_2015_131_MOESM3_ESM.pdf]

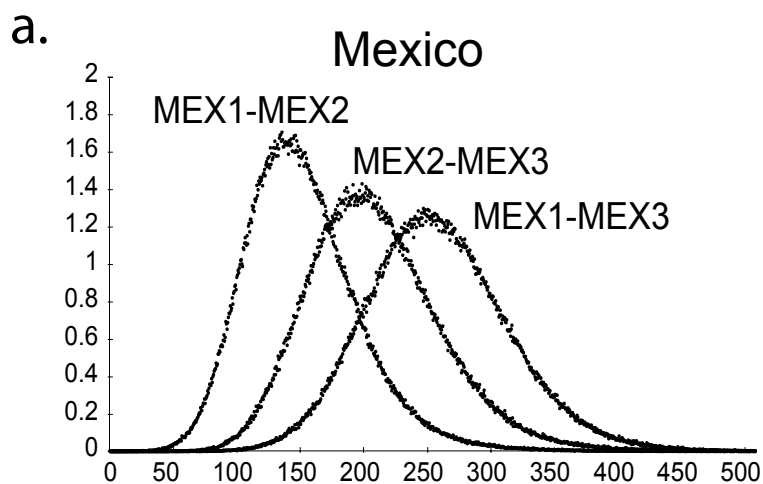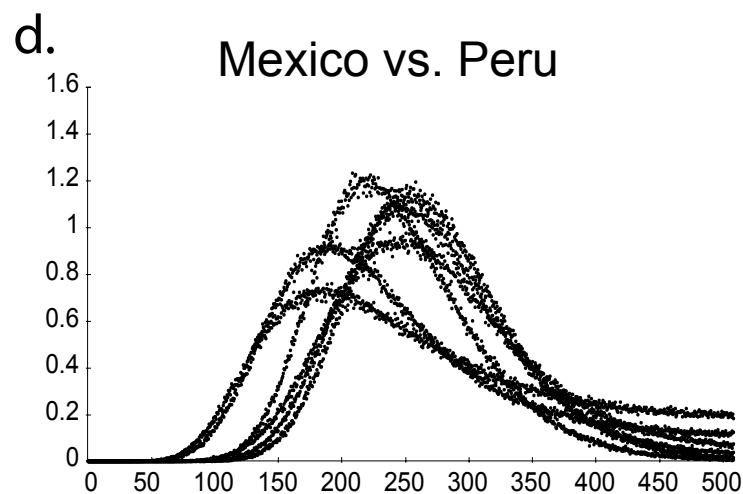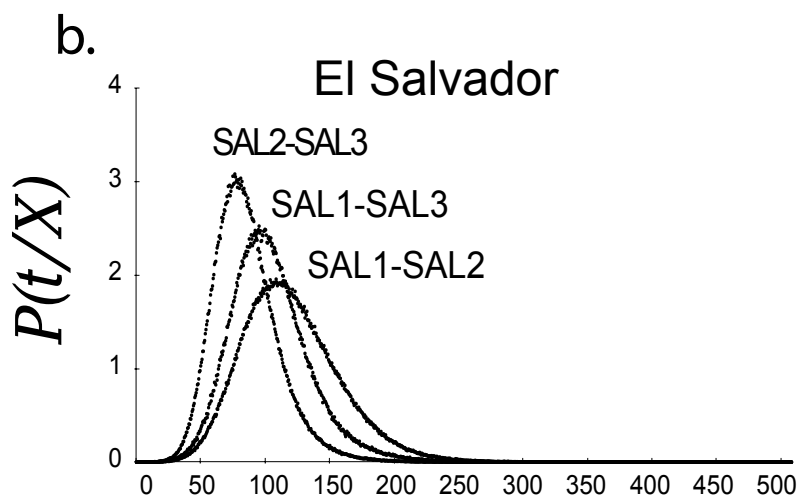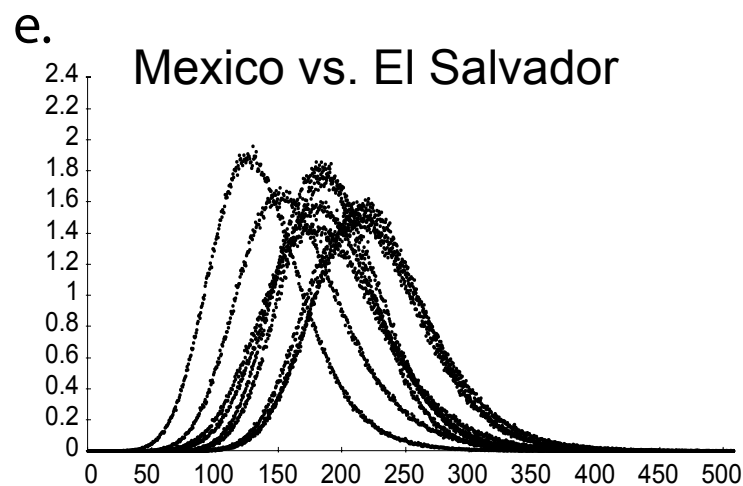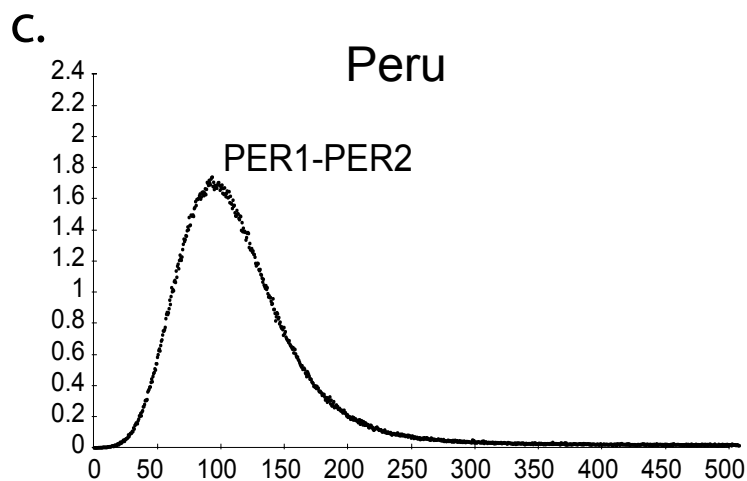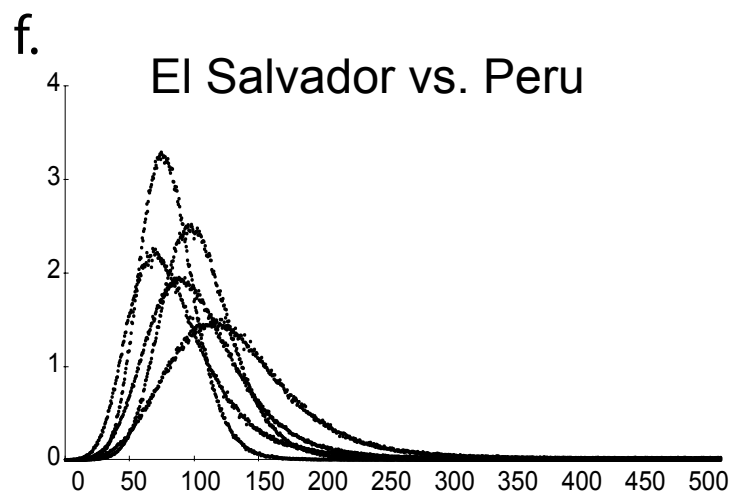

Time since divergence

Supplement: Additional file 4: — Divergence time posterior plots. Joint posterior density plots of divergence time between populations obtained from IMa2 analyses based on data from COI locus of Pollicipes elegans. (PDF 3496 kb) [file 12983_2015_131_MOESM4_ESM.pdf]

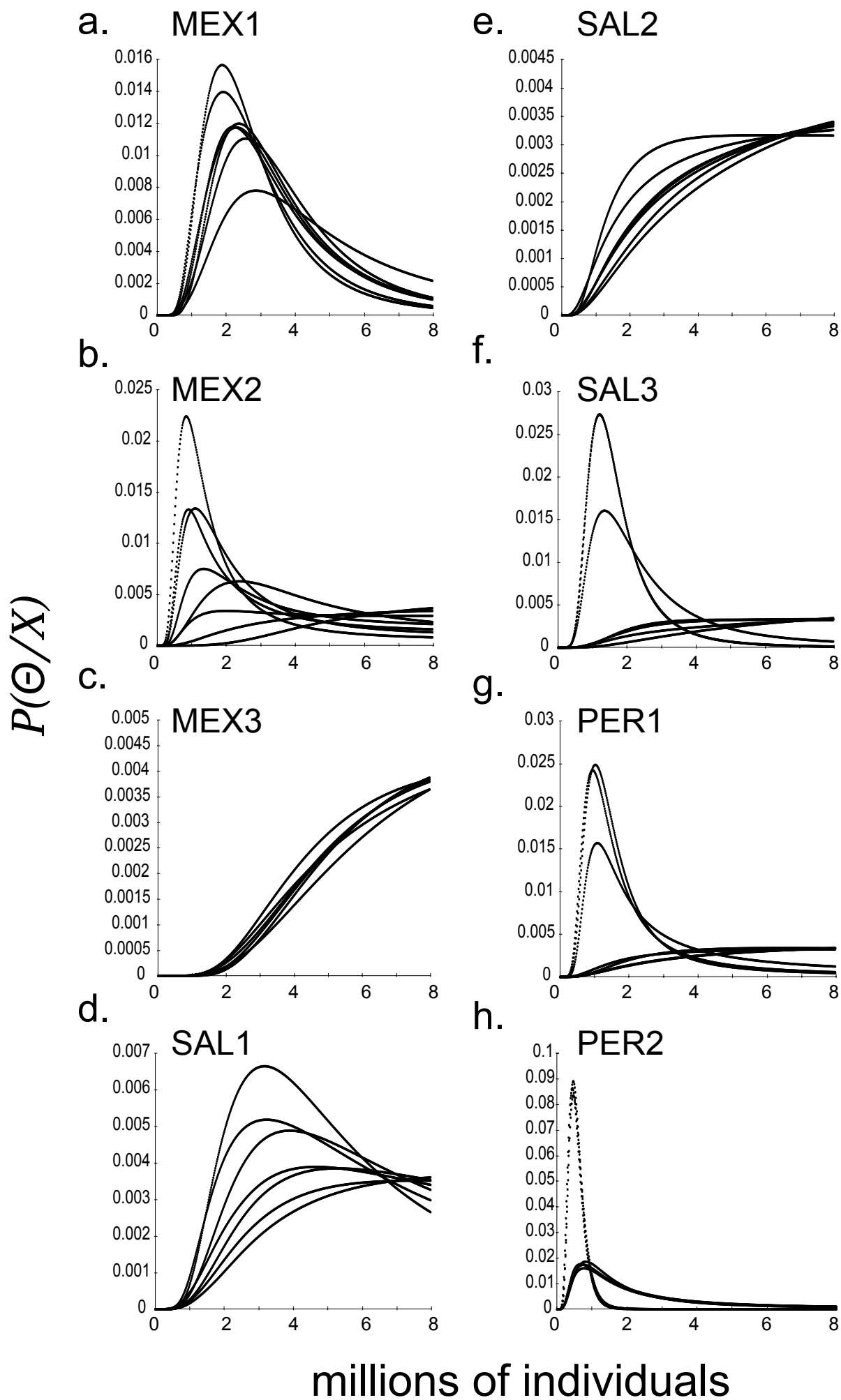

Supplement: Additional file 5: — Population size posterior plots. Joint posterior density plots of number of individuals obtained from IMa2 analyses based on data from COI locus of Pollicipes elegans. (PDF 4467 kb) [file 12983_2015_131_MOESM5_ESM.pdf]
