# Supplementary material for: Out-of-the tropics or trans-tropical dispersal? The origins of the disjunct distribution of the gooseneck barnacle Pollicipes elegans
Source: Front Zool. 2015 Dec 30;12:39. doi: 10.1186/s12983-015-0131-z (PMC4696079; doi:10.1186/s12983-015-0131-z)
Supplement: Additional file 2: — Migration rate parameter estimates. Likelihood ratio test (LLRtest) for migration rate parameters m, and population migration 2NM between pairs of populations of P. elegans. (DOCX 84 kb) [file 12983_2015_131_MOESM2_ESM.docx]

Additional file 1: Likelihood ratio test (LLRtest) for migration rate parameters *m*, and population migration *2NM* between pairs of populations of *P. elegans*.

| Population pairs^§^ | *m* | LLRtest | *2NM* | LLRtest |
| --- | --- | --- | --- | --- |
| PER1🡺PER2 | 0.02 | 0.00 | 0.00 | 0.00 |
| PER1🡸PER2 | 0.00 | 0.00 | 0.00 | 0.00 |
| MEX1🡺PER1 | 0.00 | 0.00 | 0.00 | 0.00 |
| MEX1🡸PER1 | 0.00 | 0.00 | 0.00 | 0.00 |
| MEX1🡺PER2 | 0.02 | 0.20 | 0.55 | 0.39 |
| MEX1🡸PER2 | 0.00 | 0.00 | 0.00 | 0.00 |
| MEX1🡺SAL3 | 0.00 | 0.00 | 0.00 | 0.00 |
| MEX1🡸SAL3 | 0.00 | 0.00 | 0.00 | 0.00 |
| MEX1🡺MEX3 | 0.00 | 0.00 | 0.00 | 0.00 |
| MEX1🡸MEX3 | 0.52 | **1.53^#^** | 15.31 | **1.29^#^** |
| MEX1🡺MEX2 | 0.00 | 0.00 | 0.00 | 0.00 |
| MEX1🡸MEX2 | 0.00 | 0.00 | 0.00 | 0.00 |
| MEX1🡺SAL1 | 0.00 | 0.00 | 0.00 | 0.00 |
| MEX1🡸SAL1 | 0.00 | 0.00 | 0.00 | 0.00 |
| MEX1🡺SAL2 | 0.00 | 0.00 | 0.00 | 0.00 |
| MEX1🡸SAL2 | 0.00 | 0.00 | 0.00 | 0.00 |
| SAL3🡺PER1 | 0.00 | 0.00 | 0.00 | 0.00 |
| SAL3🡸PER1 | 0.00 | 0.00 | 0.00 | 0.00 |
| JUS🡺PER2 | 0.28 | 0.18 | 0.00 | 0.00 |
| SAL3🡸PER2 | 0.00 | 0.00 | 0.00 | 0.00 |
| MEX3🡺PER1 | 0.04 | 0.08 | 2.53 | 0.24 |
| MEX3🡸PER1 | 0.00 | 0.00 | 0.00 | 0.00 |
| MEX3🡺PER2 | 0.07 | **7.10**** | 5.23 | **7.05**** |
| MEX3🡸PER2 | 0.00 | 0.00 | 0.00 | 0.00 |
| MEX3🡺SAL3 | 0.09 | **2.97*** | 6.72 | **2.89*** |
| MEX3🡸SAL3 | 0.00 | 0.00 | 0.00 | 0.00 |
| MEX3🡺SAL1 | 0.06 | 0.13 | 1.19 | 0.11 |
| MEX3🡸SAL1 | 0.00 | 0.00 | 0.00 | 0.00 |
| MEX3🡺SAL2 | 0.00 | 0.00 | 0.00 | 0.00 |
| MEX3🡸SAL2 | 0.00 | 0.00 | 0.00 | 0.00 |
| MEX2🡺PER1 | 0.04 | 0.58 | 0.53 | 0.95 |
| MEX2🡸PER1 | 0.00 | 0.00 | 0.00 | 0.00 |
| MEX2🡺PER2 | 0.06 | **2.32^#^** | 0.75 | **3.47*** |
| MEX2🡸PER2 | 0.00 | 0.00 | 0.00 | 0.00 |
| MEX2🡺SAL3 | 0.02 | 0.04 | 0.44 | 0.13 |
| MEX2🡸SAL3 | 0.00 | 0.00 | 0.00 | 0.00 |
| MEX2🡺MEX3 | 0.00 | 0.00 | 0.00 | 0.00 |
| MEX2🡸MEX3 | 0.29 | 0.27 | 0.00 | 0.00 |
| MEX2🡺SAL1 | 0.00 | 0.00 | 0.00 | 0.00 |
| MEX2🡸SAL1 | 0.00 | 0.00 | 0.00 | 0.00 |
| MEX2🡺SAL2 | 0.00 | 0.00 | 0.00 | 0.00 |
| MEX2🡸SAL2 | 0.00 | 0.00 | 0.00 | 0.00 |
| SAL1🡺PER1 | 0.00 | 0.00 | 0.00 | 0.00 |
| SAL1🡸PER1 | 0.00 | 0.00 | 0.00 | 0.00 |
| SAL1🡺PER2 | 0.57 | 0.99 | 20.00 | **1.97^#^** |
| SAL1🡸PER2 | 0.00 | 0.00 | 0.08 | 0.02 |
| SAL1🡺SAL3 | 0.00 | 0.00 | 1.47 | 0.07 |
| SAL1🡸SAL3 | 0.00 | 0.00 | 0.17 | 0.01 |
| SAL1🡺SAL2 | 0.00 | 0.00 | 0.00 | 0.00 |
| SAL1🡸SAL2 | 0.00 | 0.00 | 0.00 | 0.00 |
| SAL2🡺PER1 | 0.00 | 0.00 | 0.00 | 0.00 |
| SAL2🡸PER1 | 0.00 | 0.00 | 0.00 | 0.00 |
| SAL2🡺PER2 | 0.00 | 0.00 | 0.00 | 0.00 |
| SAL2🡸PER2 | 0.00 | 0.00 | 0.00 | 0.00 |
| SAL2🡺SAL3 | 0.00 | 0.00 | 0.00 | 0.00 |
| SAL2🡸SAL3 | 0.00 | 0.00 | 0.00 | 0.00 |

Significant values are shown in boldface.

🡸, 🡺, denote directionality of gene flow backwards in time.

**P <* 0.05, ***P <* 0.01; ^#^ inconclusive results. LLRtest= Log-likelihood ratio test. None of the results were significant after Bonferroni multiple test correction (56 comparisons *P*=0.00089). ^§^ Sampling site names as in Table 1
